# Conceptualising engagement with digital behaviour change interventions: a systematic review using principles from critical interpretive synthesis

**DOI:** 10.1007/s13142-016-0453-1

**Published:** 2016-12-13

**Authors:** Olga Perski, Ann Blandford, Robert West, Susan Michie

**Affiliations:** 1 Department of Clinical, Educational and Health Psychology, University College London, 1-19 Torrington Place, London, WC1E 6BT UK; 2 UCL Interaction Centre, University College London, 66-72 Gower Street, London, WC1E 6EA UK; 3 Cancer Research UK, Health Behaviour Research Centre, Department of Epidemiology and Public Health, University College London, 1-19 Torrington Place, London, WC1E 6BT UK

**Keywords:** Engagement, Digital, Behaviour change interventions, eHealth, mHealth, Conceptual framework, Systematic review

## Abstract

“Engagement” with digital behaviour change interventions (DBCIs) is considered important for their effectiveness. Evaluating engagement is therefore a priority; however, a shared understanding of how to usefully conceptualise engagement is lacking. This review aimed to synthesise literature on engagement to identify key conceptualisations and to develop an integrative conceptual framework involving potential direct and indirect influences on engagement and relationships between engagement and intervention effectiveness. Four electronic databases (Ovid MEDLINE, PsycINFO, ISI Web of Knowledge, ScienceDirect) were searched in November 2015. We identified 117 articles that met the inclusion criteria: studies employing experimental or non-experimental designs with adult participants explicitly or implicitly referring to engagement with DBCIs, digital games or technology. Data were synthesised using principles from critical interpretive synthesis. Engagement with DBCIs is conceptualised in terms of both experiential and behavioural aspects. A conceptual framework is proposed in which engagement with a DBCI is influenced by the DBCI itself (content and delivery), the context (the setting in which the DBCI is used and the population using it) and the behaviour that the DBCI is targeting. The context and “mechanisms of action” may moderate the influence of the DBCI on engagement. Engagement, in turn, moderates the influence of the DBCI on those mechanisms of action. In the research literature, engagement with DBCIs has been conceptualised in terms of both experience and behaviour and sits within a complex system involving the DBCI, the context of use, the mechanisms of action of the DBCI and the target behaviour.

**Electronic supplementary material:**

The online version of this article (doi:10.1007/s13142-016-0453-1) contains supplementary material, which is available to authorized users.

## INTRODUCTION

A substantial number of Internet-connected adults use some forms of digital technology to monitor or modify their health: estimates vary between 20 and 80% [1–3]. Digital behaviour change interventions (DBCIs), defined as “…a product or service that uses computer technology to promote behaviour change” [4], can, for example, be delivered through computer programs, websites, mobile phones, smartphone applications (apps) or wearable devices. Evidence suggests that DBCIs can help people change a range of different health behaviours, including smoking [5, 6], alcohol consumption [7], weight management [8], physical activity [9] and self-management of chronic conditions [10]. Some form of “engagement” with DBCIs is assumed to be important for their effectiveness [11]. A positive association between engagement and, for example, smoking cessation, weight loss and increased fruit and vegetable intake has been observed [12–14]. To date, we have not achieved a shared understanding of how to usefully conceptualise and operationalise engagement with DBCIs. This systematic review, which follows the *Cochrane Collaboration’s Handbook of Systematic Reviews of Interventions* [15], examines how engagement has been construed and measured in the behavioural science, computer science and human-computer interaction (HCI) literatures and uses this to propose an integrative definition and conceptual framework of engagement with DBCIs that can be used to generate predictions and explanations of empirical observations.

The design of DBCIs requires knowledge of intervention content, delivery, interface design and computer programming, which have traditionally been informed by separate scientific disciplines, such as behavioural science, computer science and HCI. Scientific disciplines are characterised by accumulating a body of specialist knowledge and developing a specific terminology concerned with the particular object of research [16]. Due to the multifaceted structure of DBCIs, an interdisciplinary approach, where knowledge from multiple disciplines is harnessed to develop a shared viewpoint, is required to develop a useful conceptualisation of engagement in this context [17].

Engagement has traditionally been conceptualised differently across the behavioural science, computer science and HCI literatures, which might be due to the different epistemologies subscribed to, the differing research contexts and the different objectives pursued. In the computer science and HCI literatures, engagement has traditionally been conceptualised as the subjective experience of flow, a mental state characterised by focused attention and enjoyment [18]. This kind of conceptualisation might have emerged as a result of the focus on entertainment and usability of interactive technology. In the behavioural science literature, engagement has typically been conceptualised as “usage” of DBCIs, focusing on the temporal patterns (e.g. frequency, duration) and depth (e.g. use of specific intervention content) of usage [19, 20]. This kind of conceptualisation has emerged due to the observation that while many download and try DBCIs, sustained usage is typically low [21–24]. Henceforth, two working definitions of engagement as used in the computer science and HCI literatures (“engagement as flow”) and the behavioural science literature (“engagement as usage”) are used to scope the space within which this review is conducted.

Although existing systematic reviews have assessed whether particular DBCI features (e.g. tailoring, reminders) are associated with higher engagement [25, 26] and whether engagement is associated with intervention effectiveness [11], it is not possible to synthesise results from these reviews or to draw any conclusions regarding the shape of the function (e.g. linear, non-linear) relating engagement with intervention outcomes due to the use of incomparable definitions of engagement [11]. In order to reduce fragmentation of research efforts, it would be useful to develop a shared understanding of how to conceptualise and operationalise engagement with DBCIs.

A conceptual framework can been defined as “a system of concepts, assumptions, and expectations, and the presumed relationships among them” [27]. Previous conceptual frameworks of engagement have proposed multiple interacting factors (e.g. social support, sensory appeal, ease of use) that influence “engagement as flow” or “engagement as usage” [28–30]; however, these frameworks are either not derived from empirical observations or draw only on literature from one of many interrelated scientific disciplines. For example, the framework proposed by O’Brien and Toms [28], notwithstanding its grounding in empirical observations, drew only on research from the technology literature and focused on “engagement as flow” without any links to behaviour change. Conversely, the framework by Ritterband and colleagues [29] focused on “engagement as usage” and was derived from behavioural science theory only. The model proposed by Short and colleagues [30] attempted to integrate both theoretical predictions and empirical findings from the behavioural science, persuasive design and technology literatures but did not do so in a systematic manner. Although the ontology of behaviour change interventions proposed by West and Michie provides a starting point for organising and representing DBCIs, engagement constitutes one of many important components and is hence not examined in detail [4]. It is therefore not possible to determine whether existing frameworks of engagement sufficiently explain real-world events, or whether important aspects are missing.

The aims of this review are threefold; the second and third build on output from the first:

1. To synthesise past work on engagement, addressing the following research questions:

- How has engagement been defined in the selected literatures?
- How has engagement been measured?
- What factors have been found or hypothesised to influence engagement?
- What are the proposed relationships between engagement and intervention effectiveness?

2. To develop an integrative definition of engagement with DBCIs and specify how it can be measured.
3. To develop a conceptual framework of the direct and indirect influences on engagement with DBCIs and the proposed relationships between engagement and intervention effectiveness.

## METHODS

The *Cochrane Handbook of Systematic Reviews of Interventions* [15] and the *Guidance for Undertaking Reviews in Health Care* [31] were used to inform the development of the search strategy, identify inclusion criteria, select studies and extract the data. Principles from critical interpretive synthesis (CIS) were used to inform the data synthesis [32]. As CIS is one of the few methods available that affords the synthesis of qualitative and quantitative data, it was deemed to be the most suitable method. CIS is useful when a review seeks to identify a definition of a phenomenon, as it aims to produce a higher-order structure or conceptual framework (“synthesising argument”), which is grounded in the concepts (“synthetic constructs”) identified in the reviewed articles [32]. CIS does not propose a formal method for critically appraising the quality and methodological rigour of included studies but recognises that the critical evaluation and integration of disparate forms of evidence is essentially a product of the “authorial voice” [33]. The evidence is critiqued on the basis of the implicit assumptions underlying the methodological decisions made in the reviewed articles. Hence, the quality of the evidence is considered in the development of the synthetic constructs, with the consideration based on the authors’ judgements. Principles of CIS have previously been employed in reviews of the health literature [34–36].

### Criteria for considering studies for this review

All types of study designs were included except position papers. All types of information sources were included except articles that were not peer-reviewed or not available in English. Studies with adult participants (i.e. aged 18 years or older) were included, as it was expected that different factors might influence engagement in children and adult populations due to different cognitive abilities [37]. Studies specifically targeting participants with cognitive impairment or intellectual disabilities were excluded for the same reason. DBCIs and digital interventions targeting individuals with mental health or chronic physical health conditions were included as no a priori reason suggesting that engagement should be conceptualised differently across the included topic areas could be identified. Interventions were excluded if they did not incorporate any digital component as part of the intervention itself (i.e. face-to-face delivery only) or if the technology was used solely as a tool to deliver measurement surveys. Studies involving recreational or educational digital games or multimedia software (e.g. software involving animations, sound and text) were included providing that engagement was discussed or measured. For the conceptualisation of “engagement as flow”, the games or technology did not need to be related to behaviour change. The primary outcome was definitions of engagement with DBCIs, digital games or multimedia software expressed either implicitly or explicitly. Secondary outcomes included proposed direct and indirect influences on engagement, measures of engagement and associations between engagement and intervention effectiveness expressed either implicitly or explicitly.

### Search methods for the identification of studies

#### Electronic searches

A structured search of the following electronic databases was conducted in November 2015: Ovid MEDLINE (1946—November 2015), PsycINFO (1806—November 2015), ISI Web of Knowledge (1900—November 2015) and ScienceDirect (1900—November 2015). Search terms were piloted and refined to achieve a balance between sensitivity, i.e. retrieving a high proportion of relevant articles, and specificity, i.e. retrieving a low proportion of irrelevant articles [15]. An academic librarian was consulted for the validation of the databases and the final search terms. Terms were searched for in titles and abstracts as free text terms or as index terms (e.g. Medical Subject Headings) where appropriate (see Electronic Supplementary Material 1).

#### Searching for other resources

Articles from adjacent fields not immediately or obviously relevant to the research questions were identified through expertise within the review team [32]. The Association for Computing Machinery Digital Library (a repository for conference proceedings) and relevant journals (i.e. *Journal of Medical Internet Research*, *Journal of the American Medical Informatics Association*, *Telemedicine and e-Health*) were hand searched, and reference chaining was employed to identify additional articles of interest [15, 32].

### Data collection and analysis

#### Selection of studies

Articles identified through the electronic and hand searches were merged using EndNote X7 [38] to ensure consistency. Duplicate records were removed. Two researchers independently screened (i) titles, (ii) abstracts and (iii) full texts of the identified articles against the pre-defined eligibility criteria [15]. Any disagreements were resolved through discussion and by consulting a third researcher if necessary. Inter-rater reliability was assessed based on two coding categories (i.e. inclusion versus exclusion) after the full text screening phase with the prevalence- and bias-adjusted kappa (PABAK) statistic, which controls for chance agreement [39]. The following cutoffs were used: 0.40–0.59 indicates fair agreement, 0.60–0.74 indicates good agreement and >0.75 indicates high agreement [15].

#### Data extraction and management

A pro-forma was developed by the first author to extract information about the study setting, participant characteristics, study design, data collection method and study findings [32]. The pro-forma was piloted on a sample of included articles to ensure that relevant information was captured [15]. A second researcher independently checked the pro-forma for accuracy and completeness [31]. Due to limited resources, a single reviewer completed the data extraction.

#### Quality appraisal

CIS suggests the prioritisation of seemingly relevant articles rather than favouring particular study methodologies [40]. Judgements about the relevance and underlying assumptions of articles were made by the first author and were incorporated into the data synthesis [32].

#### Data synthesis

Based on the principles from CIS, the data synthesis comprised the following steps:

1. Concepts identified in the full texts of included articles were labelled with codes by the first author. The research questions were used as a top-down coding frame; fragments of text explicitly or implicitly referring to definitions of engagement, measures of engagement, influences on engagement or associations between engagement and intervention effectiveness were coded.
2. A subsample of codes was selected through random sequence generation (https://www.random.org/) for validation by an independent researcher to increase rigour [41]. Disagreements were discussed until consensus was reached.
3. Synthetic constructs (i.e. concepts that explain similar themes) were developed from the codes, and relationships between synthetic constructs were specified by the first author.
4. The synthetic constructs and the proposed relationships between constructs were validated by an independent researcher. Disagreements were discussed until consensus was reached.
5. Two synthesising arguments (i.e. an integrative definition and its measurement, and a conceptual framework) were developed based on the synthetic constructs by the first author.
6. The synthesising arguments were refined through discussion between all co-authors.

## Results

### Summary of search results

**Fig 1:**
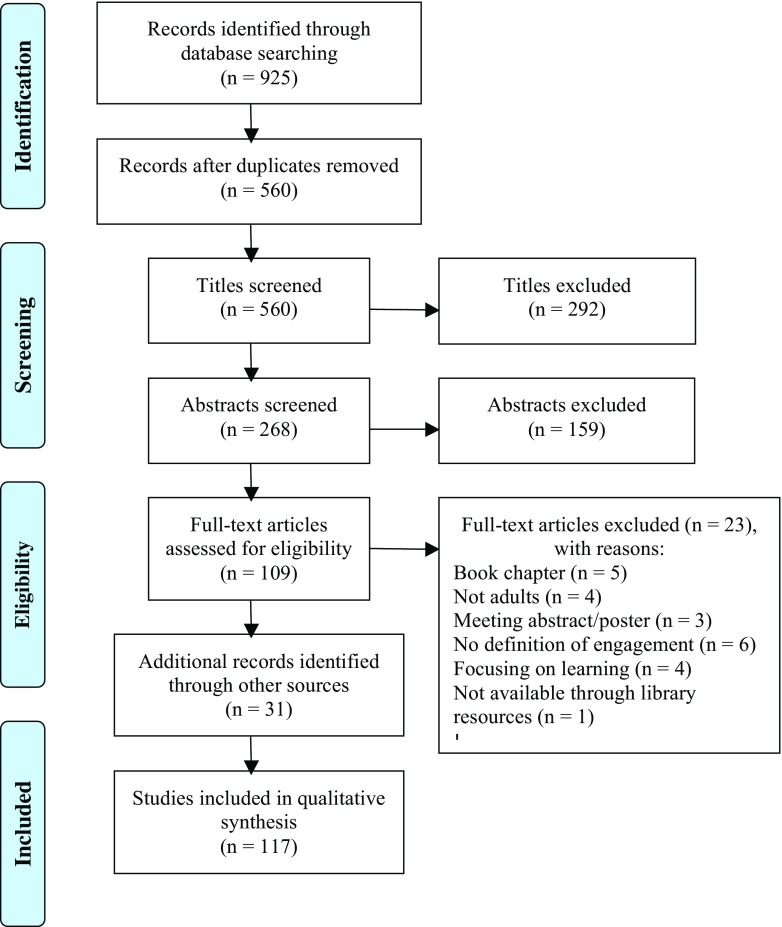
PRISMA flow diagram of the study selection process [42]

The electronic database search yielded 925 published articles. After removing duplicates, 560 articles remained for screening. A PABAK score of 0.88 was achieved after the full text screening phase, indicating high inter-rater reliability [15]. Due to this reliability score, the additional 31 information sources were screened by a single reviewer. Of the 140 full texts screened, 117 met the inclusion criteria and were included in the data synthesis. Six qualitative studies, 27 reviews, 2 mixed methods studies and 82 quantitative studies were included (see Fig. 1). Characteristics of the included studies are described in Electronic Supplementary Material 2.

### How has engagement been defined in the literature?

The following two synthetic constructs were developed: “engagement as subjective experience” and “engagement as behaviour”.

#### Engagement as subjective experience

Engagement has been conceptualised as the *subjective experience* that emerges in the momentary interaction with a system [18, 28, 43]. This kind of conceptualisation was only identified in the computer science and HCI literatures. Similarities can be found between engagement and the state of “flow”, described as a mental state characterised by focused attention, intrinsic interest and enjoyment, balance between challenge and skill, and temporal dissociation (i.e. losing track of the passage of time) [18, 44–47]. Similarities can also be found between engagement and the state of “immersion” within digital gaming, characterised by cognitive absorption, the willingness to direct emotions towards an activity and feeling cutoff from reality [43, 48–51]. As conceptual overlap was observed between these experiential qualities, the authors propose that they can be grouped under the following cognitive and emotional states: attention, interest and affect.

#### Engagement as behaviour

The majority of articles reviewed from the behavioural science literature conceptualised engagement in *behavioural* terms, suggesting that it is identical to the usage of a DBCI or its components. Engagement has further been described as the extent of usage over time [19, 52], sometimes referred to as the “dose” obtained by participants or “adherence” to an intervention [25, 53, 54], determined by assessing the following subdimensions: “amount” or “breadth” (i.e. the total length of each intervention contact), “duration” (i.e. the period of time over which participants are exposed to an intervention), “frequency” (i.e. how often contact is made with the intervention over a specified period of time) and “depth” (i.e. variety of content used) [20, 53]. In the computer science and HCI literatures, engagement has been conceptualised as the degree of involvement over a longer period of time [55], sometimes referred to as “stickiness” [56]. A distinction has also been made between “active” and “passive” engagement; while the former involves contributing to the intervention through posting in an online discussion forum, the latter involves reading what others have written without commenting, also known as “lurking” [57]. Engagement has also been conceptualised as a process of linked behaviours, suggesting that users move dynamically between stages of engagement, disengagement and re-engagement [28]. As conceptual overlap was observed between these definitions, the authors propose that engagement involves different levels of usage over time.

### Development of an integrative definition of engagement

An integrative definition of engagement with DBCIs was developed through the merging of overlapping conceptualisations as outlined above, in addition to the integration of the two overarching synthetic constructs. The following two-part definition is therefore proposed:

*“Engagement with DBCIs is (1) the extent (e.g. amount, frequency, duration, depth) of usage and (2) a subjective experience characterised by attention, interest and affect”.*

Engagement is conceptualised as a multidimensional construct: the behavioural dimensions of engagement are underpinned by the user’s subjective experience of what it feels like to be engaged with a DBCI. Engagement is considered to be a dynamic process that is expected to vary both within and across individuals over time.

### How has engagement been measured?

The following two synthetic constructs were developed: “subjective measures” and “objective measures”.

#### Subjective measures

In research settings, self-report questionnaires have frequently been used to measure engagement with digital games and DBCIs [51, 58–67]. Qualitative approaches, such as interviews or think aloud methodology, have been employed to gain a better understanding of the nature of users’ experiences of engagement with digital games and DBCIs [60, 68, 69].

#### Objective measures

Automatic tracking of use patterns, including number of logins, time spent online and the amount and type of content used during the intervention period, was the most commonly used measure of engagement in the behavioural science literature [11, 19, 20, 26, 44, 70–82]. Physiological measures including cardiac activity, respiratory depth [62] and electro-dermal activity [65], and psychophysical measures, such as eye tracking [51], have been used to measure engagement in the computer science and HCI literatures.

### Measures relating to the integrated conceptualisation of engagement

Based on the literature synthesis, we suggest that all facets of engagement proposed in the integrative definition of engagement can in principle be measured or inferred through the following: (1) user-reported interaction with the DBCI through self-report questionnaires, interview studies or think aloud studies; (2) automated recording of DBCI use (e.g. logins, page views); and (3) recording of physiological or psychophysical correlates of DBCI interaction.

### What factors have been hypothesised or found to influence engagement?

The following two synthetic constructs were developed: “context” and “DBCI”. Context was subdivided into “population” and “setting.” DBCI was subdivided into “content” and “delivery.” Relationships between constructs were specified.

### Context

#### Population

##### Psychological characteristics

*Motivation* was found to be positively associated with engagement across many studies, with none indicating a negative association [20, 68, 83–87]. As the available evidence is correlational in nature, the direction of influence cannot be assumed. It has been hypothesised that the relationship between motivation and engagement might be U-shaped; those who are least and most motivated to, for example, quit smoking, are hypothesised to disengage quickly from DBCIs due to failed and successful behaviour change, respectively [19].

*Expectations* are thought to be influential in that users are hypothesised to engage more if there is a match between their expectations and the goal of the DBCI [49, 73, 86, 88, 89]. Prior experiences of using other websites or apps, or of having tried face-to-face counselling (which may or may not have worked), might shape users’ expectations of what DBCIs can provide [90].

*Mental health*, including low mood, anxiety and stress, has been found to be negatively associated with engagement [68, 73, 87, 91–96]. A negative association with mental health was mainly observed in studies of DBCIs targeting individuals diagnosed with a mental health condition but was also observed in physical activity [68] and weight loss [94] interventions. Similarly, *experience of well-being* or believing that one does not need to work on certain issues has been found to be negatively associated with engagement [92].

*Need for cognition*, defined as the tendency to process large amounts of information [11, 30, 57, 88, 97], and *self-efficacy* to execute a given behaviour [83, 98, 99] were found to be positively associated with engagement.

*Personal relevance*, which refers to the extent to which a DBCI is perceived to apply to the individual and their particular situation, has been hypothesised to positively influence engagement [69, 78, 100–104]. Results from interview studies indicate that participants believe that lack of personal relevance is a sufficient reason for dropping out from intervention trials [86, 92, 95, 105].

##### Demographic characteristics

*Age* [20, 57, 63, 68–70, 73, 76, 79, 91, 95, 96, 99, 106–111], *gender* [20, 69, 73, 90, 95, 100, 101, 110, 111], *education* [20, 69, 91, 92, 96, 99, 106, 107, 109, 110, 112], *employment* [91, 92, 107] and *ethnicity* [57, 106] were found to be significantly associated with engagement. There was a trend towards a positive association between engagement and older age, higher educational attainment and being a woman; however, as no meta-analysis was conducted, a conclusion about the size and direction of influence cannot be drawn. *Computer literacy*, or confidence using the Internet, has been found to be positively associated with engagement [11, 20, 98, 99, 106, 108, 113]. However, as none of the included studies adequately measured baseline computer skills in their designs, a firm conclusion cannot be drawn.

##### Physical characteristics

*Physique*, including baseline weight and the presence of comorbidities, was found to be negatively associated with engagement [68, 70, 71, 91–94, 106, 112].

#### Setting

The *social* and *physical* environments in which a DBCI is used, have been hypothesised to influence engagement [4, 29, 30]. The social environment includes culture (e.g. prevailing norms), commercial environment, media and social cues. The physical environment includes financial resources, material resources, time pressure, physical cues, location, the healthcare system and policy. *Time* [86, 92, 93, 114] and *access* to hardware or the Internet [30, 115] have been hypothesised to be positively associated with engagement.

### DBCI

#### Content

DBCIs that include particular *behaviour change techniques* (BCTs), such as action plans [78], goal setting [116], feedback [59] and self-monitoring tools [78], have been found to be associated with higher engagement [78]. *Rewards* and *incentives* have been hypothesised [26, 100, 101, 117] or found [118] to positively influence engagement; however, evidence from trials in which the presence of rewards or incentives has been manipulated is scarce.

*Social support features*, referring to features that facilitate the receipt of social support, were found to positively influence engagement [76, 82, 119–124]. Features that decrease the feeling of loneliness or that increase motivation through competition with others include online discussion forums, gamification elements such as leaderboards that show users where they rank in a gamified system, and peer-to-peer contact [125, 126]. Evidence indicates that DBCIs that provide access to such features are successful in getting users who report lower social support at baseline to engage [57, 127]; however, participants who reported higher levels of social support at baseline were found to be more likely to engage with the social elements of DBCIs across a few studies [68, 86, 91, 96].

*Reminders* have been hypothesised [117, 128, 129] or found to positively influence engagement; results from a meta-analysis indicate a positive effect of reminders on engagement [130]. However, receiving too many reminders may have a negative effect on engagement due to “e-mail fatigue” [69].

#### Delivery

*Mode of delivery*, which includes face-to-face, telephone, text message, smartphone app, website and mass media delivery, has been hypothesised to influence engagement with DBCIs [4].

*Professional support features*, which include features that enable remote contact with a clinician via e-mail, telephone or text messages, have been found to positively influence engagement with DBCIs [20, 25, 26, 63, 68, 70, 73, 77, 88, 90, 95, 120, 131–134]. However, results from a randomised controlled trial (RCT) of a web-based weight loss intervention in which some participants received coaching calls from a nurse indicated that participants in the coaching arm were more likely to drop out around the time of the first coaching session, suggesting a negative influence of professional support features in particular situations [70].

*Control features*, referring to features that make users feel that they are in control of and are free to make choices about how to interact with a DBCI, have been hypothesised [51, 119] or found [52, 74, 110] to positively influence engagement. For example, results from an RCT in which participants either received content all at once or sequentially over a period of weeks suggest that participants were more likely to disengage when the content was delivered sequentially [110]. Tunnelled interventions (i.e. those that lead users through a number of predetermined steps) have been found to generate more page views compared with self-paced ones [74]. However, this may be an artefact of making users click through a pre-specified number of pages in order to progress through the DBCI.

*Novelty*, generated by regular content updates, has been found to positively influence engagement through preventing boredom [25, 26]. However, there might be a trade-off between novelty and programme *complexity*; it has been hypothesised that participants will disengage if the intervention is perceived as too long or overly complicated [26, 68, 73, 88, 131, 135, 136]. It has been hypothesised that the presence of too many features may compromise a DBCI’s *ease of use* [19], referring to whether or not it feels natural for the user to operate an interactive system. Ease of use has been hypothesised to positively influence engagement [71, 100, 137].

The *personalisation* or tailoring of content has been hypothesised [26, 52, 68, 72, 80, 103, 106, 110, 113, 119, 120, 138] or found [19, 20, 66] to positively influence engagement. *Interactivity*, referring to a two-way flow of information between a DBCI and its user, has been hypothesised [28, 48, 50, 66, 78, 100, 139] or found [19] to positively influence engagement.

*Message tone*, which refers to the terminology and wording used to communicate health messages [92, 101], and *narrative* [43, 50, 65, 103, 125, 140], referring to the presence of a storyline, have been hypothesised to positively influence engagement. Furthermore, *challenge* [61, 100, 141], *aesthetics and design* [120, 139, 142, 143] and *credibility features* [68, 73], referring to features that inculcate a feeling of trust, *familiarity* [97, 139, 144], and the provision of *guidance* or tutorials [68, 106, 145] have been hypothesised to positively influence engagement with DBCIs.

### What are the proposed relationships between engagement and the effectiveness of DBCIs?

The following four synthetic constructs were developed to explain the proposed relationships between engagement and the effectiveness of DBCIs: “mechanisms of action”, “unmeasured third variable”, “optimal dose” and “effective features”.

#### Mechanisms of action

*Mechanisms of action* proposed to mediate the effect of engagement with DBCIs on intervention effectiveness [4] include increased knowledge, motivation, affect management, cognitive restructuring, skill building [29], comprehension and practice of programme content, and increased self-efficacy [19]. A further distinction has been made between “intervention receipt”, which refers to the extent to which participants understand and can perform the skills taught, and “enactment of intervention skills”, which refers to the extent to which participants use these skills [146, 147]. It has also been hypothesised that mechanisms of action, such as accountability to a healthcare practitioner and relatedness to other individuals, might positively influence engagement with DBCIs [68, 77, 86, 96].

#### Unmeasured third variable

An *unmeasured third variable*, such as higher baseline motivation or self-efficacy, may be responsible for the observed association between increased engagement and positive DBCI outcomes. Alternatively, those who engage with DBCIs might simply be more inclined to behave healthily in general [11]. It has also been argued that the *target behaviour* itself might influence engagement [148]. For example, smokers who relapse might be more likely to stop engaging with the DBCI, while those who successfully manage their cravings might be more likely to continue engaging with the DBCI.

#### Optimal dose

*Optimal dose* refers to a pre-defined level of engagement at which specific DBCIs are effective. It has been hypothesised that the receipt of an optimal dose may explain the relationship between engagement and intervention effectiveness but that the optimal dose for particular DBCIs may vary depending on user characteristics [70, 113].

#### Effective features

The use of specific intervention features has been found to be associated with better DBCI outcomes [70]. It has been suggested that there may be a mismatch between features that participants choose to engage with frequently and *effective features* that are causally linked to intervention outcomes [104]. For example, although users may enjoy engaging with a particular feature (e.g. filling out a food diary), thus using it frequently, use of a less popular feature (e.g. “getting support” tools) might be more strongly associated with intervention outcomes, such as weight loss [70].

### Development of a conceptual framework of engagement with DBCIs

The final aim of the review was to develop a conceptual framework specifying potential direct and indirect influences on engagement and relationships between engagement and intervention effectiveness. As the framework proposed by Ritterband and colleagues [29] and the ontology proposed by West and Michie [4] explicitly linked engagement to behaviour change, we drew on these to structure our conceptual framework, mapping the other existing frameworks onto it. Additional factors identified in the reviewed literature not otherwise specified were also mapped onto the conceptual framework.

**Fig 2:**
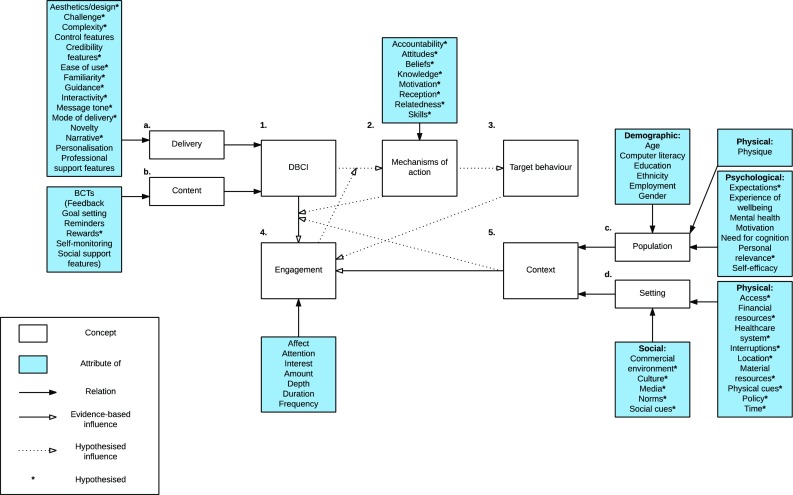
Conceptual framework of direct and indirect influences on engagement with DBCIs. Transparent boxes indicate concepts. Concepts can be defined as abstract ideas that are derived from either direct or indirect evidence [149]. Blue boxes indicate attributes of concepts. Attributes can be defined as properties that characterise a concept [150]. Solid black arrows indicate relationships between concepts and attributes. Arrows with transparent heads indicate an influence of a concept.

We propose a conceptual framework in which engagement with a DBCI influences the target behaviour through specific mechanisms of action; box 4, box 1, box 3 and box 2, respectively. Content has been found to directly influence engagement with DBCIs; box a. Delivery has been hypothesised to directly influence engagement with DBCIs; box b. The context and the target behaviour are hypothesised to directly influence engagement; box 5 and box 3, respectively. Mechanisms of action are hypothesised to indirectly influence engagement; box 2. The population (e.g. demographic, physical and psychological characteristics) has been found to directly influence engagement with DBCIs; box c. The setting has been hypothesised to directly influence engagement; box d. Engagement is hypothesised to be indirectly influenced by the moderating influence of the context on the influence of the DBCI; box 4, box 5 and box 1, respectively. Figure 2 shows this schematically. Hypothesised influences are marked with stars.

## DISCUSSION

An integrative conceptualisation of engagement with DBCIs has been developed; engagement is defined here as a multidimensional construct which can be measured through self-report questionnaires, verbal reports, automatic recording of DBCI use or recording of psychophysical manifestations. A conceptual framework was developed, which suggests that the context of use influences engagement with DBCIs either directly or indirectly by moderating the influence of the DBCI on engagement. Mechanisms of action might indirectly influence engagement and the target behaviour might directly influence engagement with DBCIs, suggesting the presence of a positive feedback loop. The proposed relationships between engagement and intervention effectiveness are tentative, as these have not been studied extensively.

The suggested behavioural and experiential dimensions of engagement can in principle be measured or inferred in every instance of a DBCI. The content, structure, length and design of specific DBCIs tend to vary, and hence, the relevance of the different dimensions of engagement will vary accordingly. Although the intended frequency, amount, duration and depth of use might be set to “1” in a one-off intervention, the individual parameters are still present and measureable. Thus, the proposed definition of engagement allows for direct comparison across different kinds of DBCIs by including multiple dimensions of engagement at its core. This has been lacking in previous conceptualisations. Evidence of higher engagement coupled with evidence of, for example, enjoyment of using a DBCI is hypothesised to predict greater DBCI effectiveness. If this is the case, the proposed definition of engagement should provide a means of generalising findings from particular DBCIs to other similar DBCIs. It may not be possible to evaluate the usefulness of the proposed definition prior to empirical work [151].

Although some self-report questionnaires designed to measure engagement demonstrate good validity and reliability [64, 152], these typically rely on measuring engagement after, as opposed to during, the event. However, the advent of new technologies allows self-reports of engagement to be measured in real-time rather than through paper-and-pencil questionnaires [153]. Although physiological measures have been used to measure engagement, notably in the HCI literature, associations between physiological and self-reported measures of engagement are weak [65]. The nature of these associations should be investigated further.

Previous conceptual frameworks have been based on theoretical predictions only or have been derived from the literature within one scientific domain [4, 28–30]. In contrast, our conceptual framework is derived from theoretical predictions and empirical observations within multiple, interrelated disciplines. This endeavour was facilitated by the use of principles from CIS, which allowed the combination of a diverse set of research findings. The proposed conceptual framework of engagement is a synthesis of existing ontologies, frameworks and models and incorporates factors not previously included. The novel components in our framework are as follows: “mental health”, “experience of well-being”, “familiarity”, “guidance” and “narrative”. The negative association between poor mental health and engagement might be explained by the observation that those with poor mental health (e.g. depression) typically experience decreased self-efficacy to, for example, stop smoking or lose weight [154, 155]. Experience of well-being might be negatively associated with engagement due to being related to the belief that one does not need any support. Familiarity with the design of DBCIs and guidance might positively influence engagement because familiar examples, design conventions or stepped how-to-use guides may inculcate feelings of comfort and ease of use. A narrative might draw users in, increasing their interest and enjoyment. Moreover, this review identified a trend towards a positive association between engagement and older age, higher educational attainment and being a woman, which merits further investigation. Although these demographic characteristics have been included in existing frameworks of engagement, the direction of influence has not been previously discussed. Through the use of a systematic, interdisciplinary approach, the proposed conceptual framework offers a comprehensive overview of the factors that may influence engagement with DBCIs and hence provides a starting point for reducing the observed fragmentation of research findings.

## LIMITATIONS

The lack of evidence supporting the claim that setting of use (e.g. culture, social norms, physical cues, healthcare pathway) directly influences engagement with DBCIs constitutes a limitation. This might either reflect the search terms used or indicate that this has not been investigated in the literature; we cannot distinguish between these explanations. There was also a lack of evidence in support of the claim that the context of use (i.e. setting and population) may moderate the influence of the DBCI on engagement. For example, the setting of use may vary depending on the mode of delivery (e.g. computer versus mobile phone). Hence, the DBCI might indirectly influence engagement through determining the setting of use; while computers may predominantly be used at home or in a clinic, mobile phones might mainly be used on the go, which may influence the amount or depth of engagement. Future research should test this hypothesis. Another limitation is that no formal quality assessment of the included articles was conducted. However, this was in line with the chosen method, which suggests that the articles should be judged on the basis of their relevance to the research question rather than their methodological rigour. This method was selected due to the conceptual nature of the research questions. A further limitation is that the data extraction and literature synthesis were conducted by a single reviewer, potentially introducing bias. Finally, the end date for the literature search (i.e. November 2015) constitutes a limitation; with the pace of technological advances and the proliferation of digital health research, it is likely that relevant literature has since been published.

## IMPLICATIONS AND AVENUES FOR FUTURE RESEARCH

The proposed integrative definition and conceptual framework of engagement with DBCIs have implications for clinical practice: the use of a shared terminology and measurement techniques will ensure more rapid advance in understanding engagement with DBCIs and developing methods to improve it. A shared conceptualisation of engagement can be used to help policymakers and commissioners to set evaluation standards for DBCIs. Moreover, the proposed conceptual framework can be used to generate testable hypotheses about how to improve engagement with DBCIs. For example, according to the conceptual framework, the presence of rewards might influence engagement with a DBCI due to increased motivation. This hypothesised link between rewards, motivation and engagement can be tested using an experimental design. Future avenues for research include the assessment of what dimensions of engagement (e.g. attention, interest, affect,  amount, duration, frequency, depth) are most strongly associated with intervention effectiveness, whether it is possible to establish benchmarks for the optimal dose of engagement across different kinds of DBCIs and whether the context of use influences engagement with DBCIs.

## CONCLUSION

Engagement with DBCIs is conceptualised here in terms of both experience and behaviour. Engagement may be influenced by the DBCI itself, the context of use, mechanisms of action of the DBCI and the target behaviour.

## Electronic supplementary material

ESM 1 (DOCX 95.9 kb)
