# Supplementary material for: Conceptualising engagement with digital behaviour change interventions: a systematic review using principles from critical interpretive synthesis
Source: Transl Behav Med. 2016 Dec 13;7(2):254–67. doi: 10.1007/s13142-016-0453-1 (PMC5526809; doi:10.1007/s13142-016-0453-1)
Supplement: Supplementary file 1 — (DOCX 95.9 kb) [file 13142_2016_453_MOESM1_ESM.docx]

**Electronic Supplementary Material 1**

Search strategy

1. “user engagement”.ti,ab,sh.

2. engag*.ti,sh.

3. immersion.ti,sh.

4. flow.ti,sh.

5. involvement.ti,sh.

6. presence.ti,sh.

7. adherence.ti,sh.

8. attrition.ti,sh.

9. 1 OR 2 OR 3 OR 4 OR 5 OR 6 OR 7 OR 8

10. digital.ti,sh.

11. web*.ti,sh.

12. computer.ti,sh.

13. online.ti,sh.

14. technology.ti,sh.

15. mobile.ti,sh.

16. smartphone.ti,sh.

17. 10 OR 11 OR 12 OR 13 OR 14 OR 15 OR 16

18. “behavior?r change”.ti,ab,sh.

19. intervention.ti,ab,sh.

20. game*.ti,ab,sh.

21. multimedia.ti,ab,sh.

22. 18 OR 19 OR 20 OR 21

23. 9 AND 17 AND 22

**Electronic Supplementary Material 2**

Table 1. Characteristics of included studies.

| **Authors (Year)** | **Country** | **Study aim** | **Population** | **Technology** | **Programme length** | **Participant characteristics** | **Study design** | **Data collection method** |
| --- | --- | --- | --- | --- | --- | --- | --- | --- |
| Al-Asadi et al. (2014) | Australia | To identify predictors of pre-treatment attrition and formal withdrawal from the Anxiety Online program. | Anxiety | Website | 12 weeks | N = 3,880;  Mean age (SD) = 36.4 (12.1);  % Female = 68.3 | Cohort | Survey |
| An et al. (2006) | US | To identify rates of participation in the RealU intervention. | Smokers | Website | 20 weeks | N = 257;  Mean age (SD) = 20.1 (1.6);  % Female = 70 | Cohort | Survey |
| Arden-Close et al. (2015) | UK | To examine patterns of web usage amongst obese primary care patients within the POWeR intervention. | Obese individuals | Website | 12 weeks | N= 132;  Mean age (SD) = 51.6 (13.0);  % Female = 66 | Secondary analysis of RCT data, intervention arm | Website logs |
| Bellg et al. (2004) | UK | To conceptualise treatment fidelity and to offer recommendations for how to incorporate fidelity measures into intervention research. | N/A | N/A | N/A | N/A | Review, narrative synthesis | N/A |
| Ben-Zeev et al. (2014) | US | To assess the usability of and engagement with a mobile phone intervention. | Serious mental illness/substance abuse | Mobile phone | 12 weeks | N = 17;  Mean age (SD) = 40.5 (11.6);  % Female = 41 | Pre- posttest | Text-message log |
| Bianchi-Berthouze et al. (2007) | UK | To understand video game engagement based on body movements. | Healthy adults | Digital game | N/A | N = 14;  Mean age (SD) = 25.0 (4.4) | Experimental, between-subjects design | Exoskeleton to measure upper body joint movement and video camera |
| Borrelli (2011) | US | To discuss the assessment, monitoring, and enhancement of treatment fidelity in public health trials. | N/A | N/A | N/A | N/A | Review, narrative synthesis | N/A |
| Bossen et al. (2013) | The Netherlands | To explore patient and study characteristics that facilitate or hinder usage of a Web-based physical activity intervention. | Patients diagnosed with hip and/or knee osteoarthritis | Website | 9 weeks | N = 199;  Mean age (SD) = 60.0 (6.3);  % Female = 63 | Mixed methods including secondary analysis of RCT data, intervention arm | Website logs and face-to-face interviews |
| Bouvier et al. (2014) | France | To gain a better understanding of what it means to be engaged and how to decide whether a behaviour reflects engagement or not. | Healthy adults | Digital game | N/A | N/A | Review, narrative synthesis | N/A |
| Boyle et al. (2012) | UK | To explore the diverse aspects of engagement and to develop a coherent understanding of engagement in computer games. | N/A | Digital game | N/A | N/A | Systematic review, narrative synthesis | N/A |
| Brigham (2015) | US | To explain the term ‘gamification’ and its current use. | N/A | N/A | N/A | N/A | Review, narrative synthesis | N/A |
| Brouwer et al. (2011) | The Netherlands | To identify methods that promote better exposure to internet interventions. | Primary prevention of physical chronic disease | Internet-delivered interventions | N/A | N/A | Systematic review, narrative synthesis | N/A |
| Brown & Cairns (2004) | UK | To develop a grounded theory of immersion. | Healthy adults | Digital game | N/A | N = 7;  % Female = 43 | Qualitative | Face-to-face interviews |
| Burns & Fairclough (2015) | UK | To quantify the degree of immersion in a digital world. | Healthy adults | Digital game | N/A | N = 20;  Mean age (SD) = 23.7 (4.2);  % Female = 35 | Experimental, mixed design | Event-related potentials to task-irrelevant stimuli |
| Cairns et al. (2013) | UK | To explore how social play influences the immersive experience of digital gameplay. | Healthy adults | Digital game | N/A | N = 24;  % Female = 42 | Experimental, within-subjects design | Questionnaires |
| Calleja (2007) | Australia | To develop a conceptual model for understanding game involvement. | N/A | Digital game | N/A | N/A | Review, narrative synthesis | N/A |
| Carter et al. (2013) | UK | To compare the acceptability of a self-monitoring weight management intervention delivered by a smartphone app with that of a website. | Overweight individuals | Smartphone app, website | 6 weeks | N = 128;  Mean age (SD) = 41.2 (8.5);  % Female = 77 | RCT | Usage data |
| Chapman, Selvarajah, & Webster (1999) | Canada | To examine engagement in two types of multimedia training systems. | Healthy adults | Interactive software | N/A | N = 72;  % Female = 69 | Experimental, between-subjects design | Questionnaires |
| Chen et al. (2015) | US | To explore the nature of engagement with an online workshop for cancer survivors. | Cancer survivors | Web-based | 8 weeks | N = 127;  Mean age (range) = 52 (26-81);  % Female = 82 | Secondary analysis of RCT data, intervention arm | Usage data |
| Chiang et al. (2011) | Taiwan | To explore online game players’ flow experiences. | Healthy adults | Digital game | N/A | N = 30;  % Female = 63 | Experimental, within-subjects design | Questionnaires |
| Chou et al. (2014) | Taiwan | To explore design factors that increase flow experience in mobile games. | Healthy adults | Mobile phone | N/A | N = 234 | Qualitative | Focus groups |
| Christensen et al. (2009) | Australia | To review rates of adherence to internet interventions for anxiety and depression. | Anxiety, depression | Internet-based interventions | N/A | N/A | Systematic review, narrative synthesis | N/A |
| Chung & Gardner (2012) | Australia | To assess the effect of different kinds of induced interruptions on players’ presence in a virtual reality theatre. | Healthy adults | Digital game | N/A | N = 36;  Mean age (SD) = 22.4 (2.9) | Experimental, mixed design | Questionnaires |
| Couper et al. (2012) | US | To explore the qualities of engagement in an online intervention designed to promote fruit and vegetable consumption. | Healthy adults | Website | 4 months | N = 2513;  Mean age = 46.3;  % Female = 69 | RCT | Usage data |
| Crutzen et al. (2012) | The Netherlands | To assess whether user control increases website use. | Healthy adults | Website | N/A | N = 668;  Mean age (SD) = 49.0 (16.0);  % Female = 49.7 | Experimental, between-subjects design | Questionnaires |
| Crutzen et al. (2013) | The Netherlands | To assess whether social presence may increase website use. | Healthy adults | Website | N/A | N = 458;  Mean age (SD) = 49.0 (16.0);  % Female = 50 | Experimental, between-subjects design | Questionnaires |
| Cugelman et al. (2011) | UK | To explore the effect of persuasive and psychological design features to inform the development of online campaigns that seek to encourage health behaviour change. | Healthy adults | Internet-based interventions | N/A | N = 6028;  % Female = 48 | Systematic review, meta-analysis | N/A |
| Cussler et al. (2008) | US | To compare weight regain in women randomised to receive an online intervention and those randomised to self-directed weight maintenance. | Overweight and obese women | Website | 12 months | N = 161;  Mean age (SD) = 48.0 (4.4);  % Female = 100, | RCT | Website logs |
| Danaher et al. (2006) | US | To describe initial patterns of participant exposure to ChewFree.com. | Smokeless tobacco users | Website | 6 weeks | N = 2523 | RCT | Website logs |
| Davies et al. (2012) | Australia | To assess the relationship between website engagement and intervention outcomes in 10,000 Steps Australia. | Healthy adults | Website | 24 months | N = 348;  % Female = 64 | Cohort | Website logs |
| Dennison et al. (2014) | UK | To assess whether POWeR intervention usage was enhanced by the addition of brief telephone coaching. | Overweight individuals | Website | 12 weeks | N = 786;  Mean age (SD) = 44.0 (12.7);  % Female = 80 | RCT | Website logs |
| Donkin et al. (2011) | Australia | To describe methods used to assess adherence to e-therapy and to evaluate the association of adherence and intervention outcomes. | Physical illness and mental health | Technology-driven interventions | N/A | N = 34,465 | Systematic review, narrative synthesis | N/A |
| Donovan et al. (2015) | US | To assess the efficacy of Wellness & Success among community and college students. | Alcohol and other drug users | Computer-based | 120 minutes | N = 415;  Mean age (SD) = 21.4 (2.2);  % Female = 73 | RCT | Website logs |
| Fang et al. (2013) | US | To develop an instrument to measure flow elements in computer gameplay. | N/A | Digital game | N/A | N/A | Instrument development and validation | Survey |
| Ferguson (2015) | UK | To examine adherence to online interventions for individuals with hearing loss. | Hearing loss | Web-based | 4 weeks | N = 44;  Mean age (SD) = 65.3 (5.7);  % Female = 34 | RCT | Website logs |
| Funk et al. (2010) | US | To examine website use patterns associated with long-term weight maintenance. | Overweight individuals at risk of cardiovascular disease | Website | 30 months | N = 348;  % Female = 63 | RCT | Website logs |
| Geraghty et al. (2013) | US | To model attrition in a dual-language internet smoking cessation intervention. | English or Spanish speaking smokers | Internet intervention | 4 weeks | N = 16430;  Mean age (SD) = 36.2 (10.7);  % Female = 47 | RCT | Survey |
| Glasgow et al. (2011) | US | To characterise usage patterns in the My Path self-management website. | Individuals with Type 2 diabetes | Website | 4 months | N = 270;  Mean age = 60;  % Female = 48 | RCT | Website logs |
| Graham et al. (2013) | US | To determine whether smokers recruited during the New Year period differed on website utilisation rates compared with smokers recruited during other time periods. | Smokers | Website | 3 months | N = 136;  Mean age (SD) = 43.2 (12.3);  % Female = 71 | Secondary analysis of RCT data | Website logs |
| Habibovic et al. (2014) | The Netherlands | To assess characteristics of ‘completers’ and ‘non-completers’ in the WEBCARE intervention. | Patients with cardioverter defibrillators | Website | 12 weeks | N = 146;  Mean age (SD) = 58.2 (9.9);  % Female = 18 | RCT | Website logs |
| Haines-Saah et al. (2015) | Canada | To determine the feasibility of engaging young adults in a user-driven, online support forum for smoking cessation. | Smokers | Web-based | 12 weeks | N = 60;  Mean age = 21 (range: 19-24);  % Female = 43 | Cohort | Manual entry of website activities |
| Han et al. (2012) | US | To assess social and psychological characteristics predictive of different levels of engagement with an online support group. | Women with a diagnosis of breast cancer | Web-based | 4 months | N = 231;  Mean age = 51;  % Female = 100 | Pre- posttest | Website logs |
| Harmat et al. (2015) | Sweden | To assess the co-variation of subjective ratings of flow with cardiovascular and respiratory responses whilst playing a computer game. | Healthy adults | Digital game | N/A | N = 77;  Mean age (SD) = 27.8 (5.4);  % Female = 52 | Experimental, within-subjects design | ECG recording, respiratory belt |
| Herbert et al. (2010) | Canada | To examine whether the Theory of Planned Behaviour and the Transtheoretical Model are able to explain adherence and attrition in an online intervention. | Chronic insomnia | Website | 5 weeks | N = 94;  % Female = 62 | RCT | Questionnaires |
| Helander et al. (2014) | Finland | To assess factors associated with sustained use of The Eatery, a mobile app that promotes healthy eating. | Individuals interested in healthy eating | Smartphone app | N/A | N = 189,770 | Cohort | Usage data |
| Henshaw et al. (2015) | UK | To explore motivations for uptake, engagement, and adherence to a computer-based auditory training programme. | Hearing loss | Computer-based | 4 weeks | N = 44;  Age range = 50-74 | Randomised, quasi-crossover | Questionnaires, focus group |
| Hilvert-Bruce et al. (2012) | Australia | To examine whether non-completers drop out due to lack of efficacy and whether changes in delivery or clinician contact improve adherence. | Anxiety, depression | Online intervention | N/A | Study 1: N = 2107;  Mean age (SD) = 40.1 (13.7);  % Female = 64  Study 2: N = 1108;  Mean age (SD) = 39.1 (13.6); % Female = 62  Study 3: N = 1090;  Mean age (SD) = 40.1 (13.8); % Female = 64 | Pre- posttest | Website logs |
| Hong et al. (2012) | Taiwan | To assess whether computer self-efficacy and ‘competitive anxiety’ are associated with flow. | Healthy adults | N/A | N/A | N = 101;  % Female = 56 | Cross-sectional | Survey |
| Horsch et al. (2015) | The Netherlands | To gain insight into strategies that enhance adherence to technology-mediated treatment. | Insomnia | Internet-based interventions | N/A | N = 2,961 | Systematic review, meta-analysis;  Qualitative | Face-to-face interviews, focus groups |
| Hsu & Lu (2004) | Taiwan | To identify predictors of users’ acceptance of online games. | Healthy adults | Digital game | N/A | N = 233;  % Female = 20 | Cross-sectional | Survey |
| Hwang et al. (2011) | Taiwan | To explore the perceived usability of video games in an elderly population. | Elderly individuals (> 60 years) | Digital game | N/A | N = 60;  % Female = 53 | Qualitative | Interviews and observation |
| Irvine et al. (2015) | US | To evaluate the efficacy of FitBack. | Individuals with non-specific lower back pain | Mobile web app | 8 weeks | N = 597;  % Female = 58 | RCT | Questionnaires |
| Jahangiry et al. (2014) | Iran | To assess adherence and attrition in a lifestyle intervention. | Individuals with metabolic syndrome | Website | 6 months | N = 160;  Mean age (SD) = 44.5 (10);  % Female = 34 | RCT | Attendance at follow-up assessment |
| Jennett et al. (2008) | UK | To assess whether immersion can be defined quantitatively. | Healthy adults | Digital game | N/A | N = 40;  Mean age (SD) = 21.0 (3.5);  % Female = 75 | Experimental, between-subjects design | Questionnaires, eye tracking |
| Jennings (2000) | US | To describe theory and research from different disciplines relevant to creating engaging websites. | N/A | Website | N/A | N/A | Review, narrative synthesis | N/A |
| Johansson et al. (2015) | Sweden | To explore participants’ experiences of non-adherence to Internet-delivered psychological treatment. | Generalised anxiety disorder | Website | 8 weeks | N = 7;  Mean age (SD) = 39.3 (17.1);  % Female = 86 | Qualitative | Face-to-face interviews |
| Kelders et al. (2012) | The Netherlands | To investigate whether particular intervention characteristics and persuasive design elements influence adherence to web-based interventions. | Health interventions | Web-based | N/A | N/A | Systematic review, narrative synthesis | N/A |
| Khadjesari et al. (2011) | UK | To determine the impact of incentives on follow-up rates in an online trial. | Alcohol users | Website | 12 months | N = 7,935;  Mean age = 38;  % Female = 57 | RCT | Questionnaires |
| Kim et al. (2013) | US | To test whether a novel mobile user engagement model may explain intention to engage. | Healthy adults | Smartphone | N/A | N = 297;  % Female = 50 | Cross-sectional | Survey |
| Klein et al. (2014) | The Netherlands | To assess the functioning of an intelligent mobile support system for therapy adherence and behaviour change. | Individuals with Type 2 diabetes, HIV, and/or cardiovascular disease | Smartphone app | N/A | N = 17 | Pre- posttest | Survey |
| Kok et al. (2014) | The Netherlands | To examine user characteristics associated with adherence to the Mobile CT programme. | Depression | Website and mobile phone | 8 weeks | N = 129 | RCT, intervention arm | Website logs |
| Kuijpers et al. (2013) | The Netherlands | To explore the possible relevance of web-based interventions aimed at increasing empowerment and physical activity in individuals with chronic illness for cancer survivors. | Chronic illness | Web-based | N/A | N/A | Systematic review, narrative synthesis | N/A |
| Lefebvre et al. (2010) | US | To develop an instrument to measure engagement with health information. | Healthy adults | Website | N/A | N = 230;  % Female = 60 | Instrument development and validation | Questionnaires |
| Leslie et al. (2005) | Australia | To describe engagement and retention with a physical activity website in a workplace setting. | Healthy adults | Website | 8 weeks | N = 655;  Mean age = 43;  % Female = 50 | RCT, intervention arm | Website logs |
| Lieberman (2006) | US | To develop and evaluate the effect of a personified guide on adherence to an online alcohol reduction programme. | Alcohol users | Website | N/A | N = 288;  Mean age (SD) = 36.0 (12.1);  % Female = 31 | RCT | Website logs |
| Lin & Wu (2014) | China | To assess the impact of SMS reminders on adherence to follow-up in digital health interventions. | Health interventions | Mobile phone | N/A | N = 12,783 | Systematic review, meta-analysis | N/A |
| Liu et al. (2009) | Taiwan | To examine user acceptance of three kinds of streaming media (text, audio, and video) during online learning. | Healthy adults | Multimedia | N/A | N = 88 | Experimental, between-subjects design | Survey |
| Ludden et al. (2015) | The Netherlands | To assess the impact of different design features on adherence to web-based wellbeing interventions. | Healthy adults | Web-based | N/A | N/A | Review, narrative synthesis | N/A |
| Mahmassani et al. (2010) | US | To examine user behaviour in a multiplayer online role-playing game. | Healthy adults | Digital game | N/A | N/A | Cohort | Game logs |
| Manwaring et al. (2008) | US | To assess whether adherence predicts outcomes in an online programme for the prevention of eating disorders. | Individuals with high levels of weight concern | Internet intervention | 8 weeks | N = 209;  % Female = 100 | RCT, intervention arm | Website logs |
| Martey et al. (2014) | US | To examine the relationships among different measures of engagement. | Healthy adults | Digital game | N/A | Study 1: N = 280;  Mean age = 21;  % Female = 59  Study 2: N = 480;  Mean age = 19.5;  % Female = 65 | Experimental, between-subjects design | Questionnaires, electro-dermal activity, mouse clicks, and mouse movement |
| McCabe & Price (2009) | Australia | To evaluate the dropout rate for an internet-based intervention for erectile dysfunction. | Erectile dysfunction | Website | 12 weeks | N = 44;  % Female = 0 | RCT | Questionnaires |
| McCambridge et al. (2011) | UK | To determine whether differences in length and relevance of follow-up questionnaires have an impact on loss to follow-up. | Alcohol users | Website | 12 months | N = 8,060 | RCT | Questionnaires |
| McClure et al. (2013) | US | To explore the effect of four design features on engagement with an internet-based smoking cessation programme. | Smokers | Website | 8 weeks | N = 1865;  Mean age (SD) = 44.2 (14.7);  % Female = 63 | Multiphase optimization strategy trial | Website logs |
| Meischke et al. (2011) | US | To determine the characteristics of parents who engage with an internet-based health intervention for their children. | Parents to children with asthma | Website | 6 months | N = 283 | RCT, intervention arm | Website logs, survey |
| Miller, Cafazzo, & Seto (2014) | Canada | To examine effective use of gamification design principles in developing mHealth apps. | Chronic illness | Smartphone app | N/A | N/A | Review, narrative synthesis | N/A |
| Mohr et al. (2013) | US | To evaluate the efficacy of telephone coaching in improving adherence to MoodManager. | Depression | Website | 12 weeks | N = 101 | RCT | Website logs |
| Morris et al. (2015) | US | To introduce and evaluate a web-based, peer-to-peer cognitive reappraisal platform. | Depression | Website | 3 weeks | N = 166;  Mean age (SD) = 23.7 (5.3);  % Female = 72 | RCT | Website logs, questionnaires |
| Morrison & Doherty (2014) | UK | To conduct an exploration of the use of visualisations of log data to improve understanding of engagement with web-based interventions. | Depression | Website | N/A | N = 326 | Secondary analysis of cohort data | Website logs |
| Morrison et al. (2014) | UK | To examine the effect of two different design features (tailoring and self-assessment) on engagement. | Mild bowel problems | Website | N/A | Study 1: N = 24;  Median age = 25;  % Female = 67  Study 2: N = 178;  Mean age (SD) = 30.2 (11.7);  % Female = 78 | Qualitative  Partial factorial design | Interviews  Website logs |
| Murray et al. (2013) | UK | To assess whether adherence and retention are related. | Alcohol users | Website | 12 weeks | N = 7,932;  Mean age (SD) = 38.0 (11.0);  % Female = 57 | Secondary analysis of RCT data | Website logs, questionnaires |
| Neve et al. (2010) | Australia | To describe the prevalence and predictors of dropout and non-usage attrition in a web-based weight loss programme. | Overweight individuals | Website | 12 months | N = 9,599;  Mean age (SD) = 35.7 (9.5);  % Female = 86 | Cohort | Website logs |
| Nicholas et al. (2010) | Australia | To explore reported reasons for non-adherence to an online psycho-education programme. | Bipolar disorder | Website | 8 weeks | N = 39;  % Female = 56 | RCT;  Qualitative | Website logs;  Interviews |
| O’Brien & Toms (2008) | Canada | To conceptually and operationally define engagement with technology. | Healthy adults | Technology | N/A | N = 17;  % Female = 59 | Review, narrative synthesis;  Qualitative | Face-to-face interviews |
| O’Brien & Toms (2010) | Canada | To develop an engagement scale. | Healthy adults | Website | N/A | Study 1: N = 440;  % Female = 69  Study 2: N = 802;  % Female = 70 | Instrument development and validation | Questionnaires |
| Oh & Sundar (2015) | US | To explore the effect of two different interactivity types (modality and message) on website engagement. | Healthy adults | Website | N/A | N = 167;  Mean age = 19.6;  % Female = 58 | Experimental, between-subjects design | Questionnaires |
| Oinas-Kukkonen & Harjumaa (2009) | Finland | To describe a framework for the design and evaluation of Persuasive Systems. | N/A | N/A | N/A | N/A | Review, narrative synthesis | N/A |
| Park et al. (2010) | US | To examine the effect of exposure to a pre-game story on the feeling of presence during gameplay. | Healthy adults | Digital game | N/A | Study 1: N = 30;  % Female = 80  Study 2: N = 24;  % Female = 58 | Experimental, between-subjects design | Questionnaires |
| Parks (2014) | US | To outline important design considerations in online positive psychological interventions. | Healthy adults | Website | N/A | N/A | Review, narrative synthesis | N/A |
| Peels et al. (2012) | The Netherlands | To assess user characteristics associated with participation and attrition in web-based and print-based tailored physical activity interventions. | Aging population | Website | 12 weeks | N = 1,729; Mean age = 48.3  % Female = 52 | Cluster RCT | Questionnaires |
| Poirier & Cobb (2012) | US | To examine the association between social ties and engagement with a health and wellness online intervention. | Healthy adults | Website | 4 weeks | N = 84,828;  % Female = 84. | Cohort | Website logs |
| Postel et al. (2011) | The Netherlands | To examine attrition prevalence and pre-treatment predictors of attrition in a sample of open-access users of a Web-based program. | Problem drinkers | Website | 3 months | Study 1: N = 780;  Mean age (SD) = 47.5 (10.8); % Female = 54  Study 2: N = 144;  Mean age (SD) = 45.8 (9.7);  % Female = 58 | Cohort (Study 1), RCT (Study 2) | Website logs |
| Richardson et al. (2010) | US | To measure the effect of adding online community features to an Internet-based walking program on attrition and average daily step counts. | Sedentary adults | Website | 16 weeks | N = 324;  Mean age (SD) = 52.0 (11.4); % Female = 66 | RCT | Step counts, website logs |
| Richardson et al. (2013) | US | To examine the effectiveness of a Web-based smoking cessation intervention, to identify the most effective features, and to gain insight into who is most likely to use those features. | Smokers | Website | 1 month | N = 1,033, % Female = 52 | Cohort | Website logs |
| Ritterband et al. (2009) | US | To propose a model to help guide future development of online interventions and to predict and explain behavior change afforded by online interventions. | N/A | Internet interventions | N/A | N/A | Review, narrative synthesis | N/A |
| Sainsbury et al. (2015) | Australia | To assess the acceptability of an online intervention to improve diet adherence in coeliac disease and to examine the relationships with participant characteristics, attrition, and effectiveness. | Coeliac disease | Website | N/A | N = 189 | RCT | Completion of follow-up assessment |
| Schønau-Fog & Bjørner (2012) | Denmark | To propose a method that can be used to empirically investigate the experience of wanting to continue playing. | Healthy adults | Digital game | N/A | N = 30 | Qualitative | Interviews |
| Schubart et al. (2011) | US | To review what factors influence user engagement in Internet-based behavioral interventions for chronic illness. | Chronic illness | Internet-based interventions | N/A | N/A | Systematic review, narrative synthesis | N/A |
| Schwarzer & Satow (2012) | Germany | To predict smoking abstinence in internet users who engage with a virtual community. | Smokers | Virtual community | 10 weeks | N = 13,174 | Cohort | Website logs |
| Sharek & Wiebe (2014) | US | To investigate a novel technique for measuring video game engagement by capturing behavioral data without interfering with the main task. | Healthy adults | Digital game | N/A | N = 156;  Mean age (SD) = 30.8 (10.2);  % Female = 58 | Experimental, between-subjects design | Game-clock clicks |
| Shaw et al. (2014) | US | To describe how fidelity recommendations may be applied in mobile phone interventions for weight loss. | Overweight individuals | Mobile phone | N/A | N = 261 | Review, narrative synthesis | N/A |
| Short et al. (2015) | Australia | To propose a new model of user engagement that can be used to guide the development and evaluation of online behaviour change interventions. | N/A | Online interventions | N/A | N/A | Review, narrative synthesis | N/A |
| Stark et al. (2011) | US | To describe dietary self-monitoring rates among participants randomised to the intervention arms of two pilot studies. | Dialysis patients | Electronic diary | 16 weeks | Study 1: N = 22;  Mean age = 56;  % Female = 40  Study 2: N = 26;  Mean age = 52;  % Female = 44 | RCT, intervention arm | Website logs |
| Steinberg et al. (2014) | US | To examine patterns and predictors of self-monitoring adherence and the association between adherence and weight change in an online intervention. | African-American women with low income | Interactive voice response | 12 months | N = 185;  Mean age (SD) = 35.4 (5.5)  % Female = 100 | RCT | IVR completion |
| Strecher et al. (2008) | US | To determine whether engagement in a web-based smoking cessation intervention predicts 6-month abstinence, whether particular groups are more likely to engage, and whether particular components influence engagement. | Smokers | Website | 6 months | N = 1,866;  Mean age = 46.3;  % Female = 60 | Fractional factorial design with 16 arms | Website logs |
| Ubhi et al. (2015) | UK | To conduct a preliminary evaluation of the effectiveness of a novel smoking cessation smartphone application. | Smokers | Smartphone application | 28 days | N = 1,170  % 16-29 years = 50.4  % 30-49 years = 45.4  % 50+ years = 4.2  % Female = 64.5 | Observational prospective cohort | Automated recording logins, time spent, page views |
| VanDeMark et al. (2010) | US | To describe the characteristics of participants in the E-TREAT intervention, and to examine the characteristics that predict active engagement. | Substance use disorder | Website | 3 months | N = 157;  Mean age (SD) = 36.6 (9.7);  % Female = 52 | Cohort | Contact log |
| Van den Berg et al. (2006) | The Netherlands | To assess engagement with an Internet-based physical activity intervention with individual supervision. | Rheumatoid arthritis | Web-based | 12 months | N = 82;  Median age (IQR) = 49.5 (12.9);  % Female = 76 | RCT, intervention arm | Website logs |
| Vandelanotte et al. (2007) | Australia | To review outcomes of web-based physical activity interventions and to identify relationships of intervention components with behavioural outcomes. | Healthy adults | Website | N/A | N = 4,845 | Systematic review, narrative synthesis | N/A |
| Voils et al. (2014) | US | To present approaches to inform intervention duration, frequency, and amount when 1) the researcher has no a priori expectation, and 2) when the researcher does have an a priori expectation. | N/A | N/A | N/A | N/A | Review, narrative synthesis | N/A |
| Wang et al. (2012) | US | To examine the mediating role of adherence to self-monitoring of diet and physical activity on weight loss in an online trial. | Overweight individuals | Web-based | 12 months | N = 210;  Mean age (SD) = 46.8 (9.0);  % Female = 85 | RCT | Usage data |
| Wanner et al. (2010) | Switzerland | To assess and compare user characteristics and adherence to Active Online in an open access context over time and between trial participants and open access users. | Healthy adults | Website | 6 weeks | Study 1: N = 836;  Mean age = 43.1;  % Female = 75  Study 2: N = 5,084;  Mean age = 38.4;  % Female = 50 | RCT (Study 1), cohort (Study 2) | Website logs |
| Webber et al. (2008) | US | To examine the relationships between motivation, adherence, and weight loss in an online behavioral weight-loss intervention. | Overweight individuals | Website | 16 weeks | N = 66;  Mean age (SD) = 50.1 (9.9);  % Female = 100 | RCT | Website logs |
| West & Michie (2016) | UK | To provide guidance on the development and evaluation of digital behaviour change interventions in healthcare. | N/A | N/A | N/A | N/A | Review | N/A |
| Weston et al. (2015) | UK | To investigate measurements of engagement using a health-based quiz app. | Healthy adults | Smartphone app | N/A | N = 29;  Age range= 21-56;  % Female = 59 | RCT | Usage data |
| Whiteside et al. (2014) | US | To get user feedback on messaging content intended to engage suicidal individuals. | Suicidal individuals | Web-based | N/A | N = 34;  % Female = 68 | Cross-sectional | Survey |
| Zhou (2013) | China | To identify factors associated with the initial adoption of mobile games. | Healthy adults | Mobile phone | N/A | N = 231  % Female = 37 | Cross-sectional | Survey |
